# Muscle Carnitine Palmitoyltransferase II (CPT II) Deficiency: A Conceptual Approach

**DOI:** 10.3390/molecules25081784

**Published:** 2020-04-13

**Authors:** Pushpa Raj Joshi, Stephan Zierz

**Affiliations:** Department of Neurology, Martin-Luther-University Halle-Wittenberg, Ernst-Grube Str. 40, 06120 Halle (Saale), Germany; stephan.zierz@uk-halle.de

**Keywords:** CPT II, muscle, rhabdomyolysis, fatty acids, mutation, Genotype-Phenotype

## Abstract

Carnitine palmitoyltransferase (CPT) catalyzes the transfer of long- and medium-chain fatty acids from cytoplasm into mitochondria, where oxidation of fatty acids takes place. Deficiency of CPT enzyme is associated with rare diseases of fatty acid metabolism. CPT is present in two subforms: CPT I at the outer mitochondrial membrane and carnitine palmitoyltransferase II (CPT II) inside the mitochondria. Deficiency of CPT II results in the most common inherited disorder of long-chain fatty acid oxidation affecting skeletal muscle. There is a lethal neonatal form, a severe infantile hepato-cardio-muscular form, and a rather mild myopathic form characterized by exercise-induced myalgia, weakness, and myoglobinuria. Total CPT activity (CPT I + CPT II) in muscles of CPT II-deficient patients is generally normal. Nevertheless, in some patients, not detectable to reduced total activities are also reported. CPT II protein is also shown in normal concentration in patients with normal CPT enzymatic activity. However, residual CPT II shows abnormal inhibition sensitivity towards malonyl-CoA, Triton X-100 and fatty acid metabolites in patients. Genetic studies have identified a common p.Ser113Leu mutation in the muscle form along with around 100 different rare mutations. The biochemical consequences of these mutations have been controversial. Hypotheses include lack of enzymatically active protein, partial enzyme deficiency and abnormally regulated enzyme. The recombinant enzyme experiments that we recently conducted have shown that CPT II enzyme is extremely thermoliable and is abnormally inhibited by different emulsifiers and detergents such as malonyl-CoA, palmitoyl-CoA, palmitoylcarnitine, Tween 20 and Triton X-100. Here, we present a conceptual overview on CPT II deficiency based on our own findings and on results from other studies addressing clinical, biochemical, histological, immunohistological and genetic aspects, as well as recent advancements in diagnosis and therapeutic strategies in this disorder.

## 1. Long-Chain Fatty Acids

Long-chain fatty acids (lcFA) are important sources of energy, especially for the heart, liver and muscles. They are used as the preferred substrate in the myocardium at rest and during prolonged exercise in skeletal muscle [1]. Additionally, fatty acids also serve as building blocks for membrane lipids and cellular signaling molecules [2,3,4,5]. Ketone bodies produced in the liver during the oxidation of long-chain fatty acids can replace glucose in the brain and thus ensure the maintenance of normal content of sugar in the blood. In addition, the ketone bodies prevent the breakdown of muscle protein for the purpose of gluconeogenesis [6]. Oxidation of fatty acids takes place in mitochondria. The transport of fatty acids from cytoplasm to mitochondria occurs through long-chain fatty acid transport system.

## 2. Long-Chain Fatty Acid Transport System

The enzyme carnitine palmitoyltransferase (CPT) facilitates the transfer of long-chain fatty acids from cytoplasm into mitochondria during the oxidation of fatty acids [7]. CPT is present in two sub-forms, CPT I and CPT II, that are localized in mitochondria [8,9]. The transmembrane protein CPT I is located at the outer mitochondrial membrane and CPT II is located inside the mitochondria [10].

**Figure 1:**
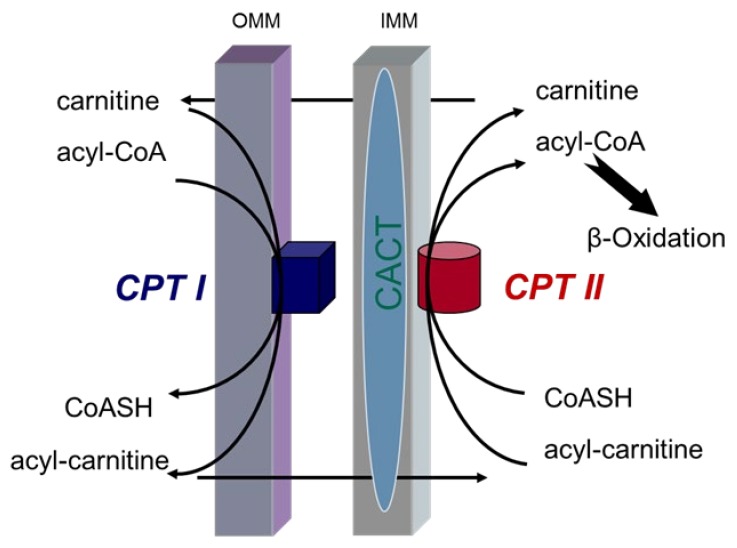
Transport system for esterification of fatty acids through mitochondrial membranes (OMM: outer mitochondrial membrane; IMM: inner mitochondrial membrane, CPT I & II: carnitine palmitoyltransferase I and II; CACT: Carnitine- Acylcarnitine-Translocase; CoASH: free Coenzyme A).

CPT I and CPT II along with Carnitine-Acylcarnitine-Translocase (CACT) play a vital part in the transport system for esterification of fatty acids through the mitochondrial membrane (Figure 1). CPT I at the outer mitochondrial membrane catalyzes the first transport step. At this step, long-chain acyl-CoA along with carnitine are converted into long-chain acyl carnitine and coenzyme A. This catalysis step and all subsequent reactions including β-oxidation are rate-limiting [11]. The transesterified acylcarnitines are then transported from cytosol into intermembrane space [12,13]. One of the products of this reaction—acyl carnitine—is then transported through the inner mitochondrial membrane by CACT. The remaining acyl of the acyl carnitine is transferred back to coenzyme A on the inner mitochondrial membrane by CPT II that catalyzes the reverse reaction by transferring the acyl group from carnitine to acyl-CoA. Thus, produced acyl-CoA is then available for β-oxidation [11]. The carnitine released in this step returns back into the intermembrane space of the mitochondrion through the CACT and is available for the re-transport of fatty acids (Figure 1).

To better understand the localization and functions of CPT I, CPT II and CACT, different studies using cross-linking reagent, blue native electrophoresis, immunoblotting, and mass spectrometry have been performed. On the basis of these studies, CPT I is suggested to form hetero-oligomeric complexes with long-chain acyl-CoA synthetase (ACSL) and voltage-dependent anion channel (VDAC). This confirms that CPT I is an integral part of an outer membrane fatty acid complex [14]. Moreover, CPT II and CACT have been demonstrated to interact in forming a supramolecular complex in the inner mitochondrial membrane that facilitates channeling the acylcarnitines [13].

A disorder of the CPT system may affect both CPT I and CPT II enzyme production. Both of the resulting rare diseases follow an autosomal recessive mode of inheritance.

## 3. Carnitine Palmitoyltransferase II (CPT II) Deficiency

CPT II deficiency is the most common inherited disorder of the rare long-chain fatty acid oxidation defects. Extra energy is required during strenuous conditions such as prolonged exercise, fasting, exposure to cold, fever and emotional stress. The extra energy demand in these conditions is fulfilled by oxidation of fatty acids. As discussed above, long-chain fatty acids need to be trans-esterified to acylcarnitine for their subsequent oxidation inside the mitochondria. CPT I, CPT II and CACT play integral role in transport of fatty acids from cytosol into mitochondria [15].

## 4. Three Phenotypes of CPT II Deficiency

(i) Lethal neonatal form presents with hypoketotic hypoglycaemia and severe hepatomuscular symptoms [16,17,18,19]. It is characterized by reduced CPT II enzyme activity in multiple organs, reduced carnitine concentration and increased concentrations of long-chain acylcarnitines and lipids in serum. The patients suffer from liver failure, hypoketotic hypoglycaemia, cardiomyopathy, respiratory distress, and/or cardiac arrhythmias. Affected individuals are reported with liver calcifications and cystic dysplastic kidneys [16,17]. So far, about 20 families with the lethal neonatal form [19,20,21,22,23] have been described. CPT II deficiency is almost undetectable during pregnancies as it causes severe cerebral malformations of the foetus. Hence prevalence of this form seems to be higher than previously suspected [24].

(ii) Severe infantile hepatocardiomuscular form is characterized by hypoketotic hypoglycaemia, liver failure, cardiomyopathy, and peripheral myopathy [16,25,26,27]. The main cause of causality during infancy is reported to be cardiac arrhythmia [16,28]. Apart from that, hepatomegaly [27] and Dandy-Walker malformation are also reported to be fatal [26]. So far, some 30 families with this form are described [16,25,26,27,28].

(iii) The classical muscle form is rather mild and it is clinically characterized by recurrent episodes of muscle pain, muscle weakness, and rhabdomyolysis triggered mostly by prolonged exercise [24,29,30,31,32]. Affected individuals generally do not have muscle weakness in between the attacks. Some individuals have only a few severe attacks and are asymptomatic most of their lives, whereas others have frequent myalgia, even after moderate exercise related to daily activities [33]. End-stage renal disease due to interstitial nephritis with acute tubular necrosis requiring dialysis is also occasionally reported [34].

## 5. Muscle Form of CPT II Deficiency

Muscle form can manifest from infancy to adulthood (OMIM 600650). It is sometimes also termed as ‘adult form’ [8,11,13]. However, cases with early childhood manifestation have also been reported [24,35,36,37,38,39,40,41,42]. On the other hand, adult patients with severe hepatocardiomuscular infantile form of CPT II deficiency are also sporadically reported [21,42].

### 5.1. Clinical Presentations

In muscle CPT II deficiency, onset is seen generally in childhood or early adulthood [29,43]. Single or multiple attacks of severe myalgia (often with myoglobinuria) or frequent exercise-induced myalgia are more common symptoms [31,39,40,43,44]. The most important trigger factor for attacks is reported to be exercise [31,39,40,43,44]. Usually no signs of myopathy (weakness, myalgia, elevated serum creatine kinase (CK)) are seen between attacks. Even during the attacks, the severity of pain is highly variable and some of these attacks may be complicated by acute renal failure [33]. Single cases with permanent weakness are also reported [44,45]. In general, the CK levels in between the attacks are within the reference range [20]. However, permanent elevation of serum CK is observed in approximately 10% of affected individuals [46].

More than 75% patients reported so far are males. This shows a clear male predominance in muscle CPT II deficiency [29,43,47,48,49]. It is not clear, however, whether the male predominance is due to sex-related differences in exercise activities, an X-chromosomal modifier gene, or hormonal factors such as oestrogen that seem to be a regulator of CPT [50].

### 5.2. Biochemical Features

Previously, using the isotope forward assay under optimal conditions and in the presence of 0.2mM malonyl-CoA and 0.4% Triton X-100, we have shown that the total enzyme activity (CPT I+II) was not significantly different in patients and healthy controls. However, the residual CPT II activity upon inhibition by malonyl-CoA (highly regulated molecule in fatty acid synthesis that inhibits the rate-limiting step in beta-oxidation of fatty acids) and Triton X-100 (a common non-ionic surfactant and emulsifier that solubilize proteins) was significantly decreased in patients compared to that in controls [29,46]. These observations reflect that CPT II deficiency is not exclusively due to loss of total enzyme activity but rather due to abnormal regulation of the enzyme. Significance of malonyl-CoA and Triton X-100 in these experiments reflects that exposure to these and similar substances would trigger symptoms in CPT II deficient patients. Mainly, Triton X-100 is used in manufacturing of different biopharmaceutical products, plating of metals as well as ingredient in some vaccines (influenza). Apart from that, it is widely found in variety of cleaning compounds used for everyday purposes.

Different studies have postulated a significant loss of CPT II activity in muscle homogenates of patients. Backward assay method using 2 mM palmitoyl-l-carnitine is used to measure total CPT activity in most of these studies [51,52,53]. However, as low as 0.2 mM dl-palmitoylcarnitine is already known to progressively inhibit enzyme activity in both patients and controls [54]. This observation reflects that a reduced CPT II protein activity could be interpreted by abnormal inhibition, supporting the hypothesis of an abnormal regulated enzyme [29,54].

### 5.3. Pathobiochemical Characteristics

In muscle CPT II deficiency, acylcarnitine is only partially transported across the inner mitochondrial membrane and hence the conversion of acylcarnitine into acyl-CoA is insufficient. This results is an accumulation of acylcarnitine in the plasma. Concentration of acylcarnitine in plasma can be used for diagnostic purposes. High-performance liquid chromatography tandem mass spectrometry of dried blood spots demonstrates an elevation of Dodecanoyl-L-carnitine (C12) to Octadecanoyl-L-carnitine (C18) acylcarnitines, notably of Hexadecanoyl-L-carnitine (C16) and Octadecenoyl-L-carnitine (C18:1) in muscle CPT II deficiency. However, CPT II deficiency cannot be excluded based solely on acylcarnitine quantification in dried blood spots alone and investigation of plasma is recommended for reliable diagnosis [55]. In our experience there are numerous false negative results based on dried blood analysis.

An MRI study on patients with long-chain fatty acid (lcFA) disorder showed association of specific patterns of increased T1 weighted (T1W) and Short-TI Inversion Recovery (STIR) signal intensity. These patterns reflect lipid accumulation and inflammation secondary to lcFA defects and progressive muscle damage. T1W and STIR signal intensities were less prominent in MRIs of muscle CPT II deficient patients [56]. However, the significance of MRI investigation in muscle CPT II deficiency is still unclear.

**Figure 2:**
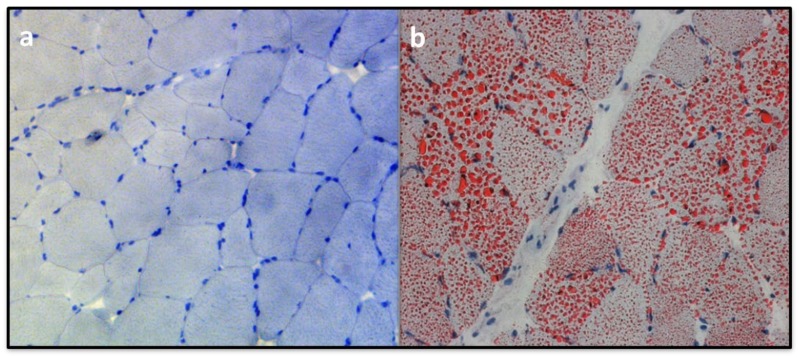
Sudan staining of muscle biopsy sections (200× magnifications) of (**a**) CPT II deficient patient compound heterozygous for p.Ser113Leu/p.Arg151Gln mutations and (**b**) primary carnitine deficient patient. Red dots represent lipid accumulation.

In muscle CPT II deficiency, normal or very slightly increased content of lipid is seen in biopsy sections. This is similar in some other lipid metabolism disorders such as, very-long-chain acyl-CoA dehydrogenase (VLCAD) deficiency, mitochondrial trifunctional protein (MTP) deficiency and phosphatidic acid phosphatase deficiency [57]. Additionally, the histological examination of the muscle biopsy sections of muscle CPT II deficient patients seldom show accumulation of lipid droplets in Sudan staining (Figure 2).

Sudan staining of muscle biopsy section of a 35-year-old female muscle CPT II deficient patient compound heterozygous for p.Ser113Leu/p.Arg151Gln mutations (Figure 2A) was compared with that of an adult patient with primary carnitine deficiency (Figure 2B). The CPT II deficient patient conveyed of suffering from at least 50 attacks per year. In between the attacks, the patient did not experience severe physical disability. The residual CPT II activities upon malonyl CoA and Triton X-100 were severely reduced in muscle biopsy that was taken during attack free interval. This shows that the symptoms in muscle CPT II deficiency are also only intermittent in comparison to other lipid accumulation deficiencies such as carnitine deficiency or neutral lipid storage disease [57,58]. Hence, the normal protein content and enzyme activity seem to allow a normal function of the CPT system in situations without stress on fatty acid metabolism.

### 5.4. Immunohistochemistry

**Figure 3:**
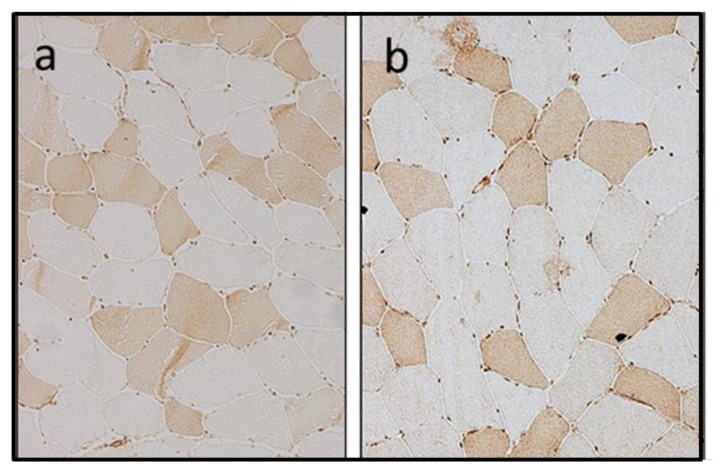
Immunohistochemical staining of CPT II (200× magnification) (**a**) control muscle section and (**b**) muscle section of CPT II deficient patient (Figure adapted with kind permission of the authors from Lehmann & Zierz; 2014).

Immunohistological analysis of muscle sections of CPT II deficient patients has demonstrated CPT II in similar intensity as in controls (Figure 3). The myosin heavy chain (MHC)-slow staining immunoreactivity was expressed predominantly in type I fibres both in the muscles of CPT II deficient patients and of controls [59]. This observation strengthens the notion that in muscle CPT II deficiency enzyme activity and protein content are not reduced, but are rather abnormally inhibited during stress and increased fatty acid metabolism.

### 5.5. Molecular Genetic Aspects

In patients with the muscle CPT II deficiency, a common p.Ser113Leu mutation is identified in about 70% of mutant alleles [29,31,32,40]. The phenotypes of this mutation are generally mild. This mutation is exclusively associated with muscle form of CPT II deficiency. Furthermore, around 100 other rare disease causing mutations that are associated with muscle CPT II deficiency have been reported [29,60]. Mutations are reported in all five exons of *CPT2* gene. However, most of the mutations are located in exon 4 (Table 1). The biochemical consequences of these mutations are still controversial [61]. Hypotheses include lack of enzymatically active protein, partial enzyme deficiency and abnormally regulated enzyme [62]. In previous studies, CPT activities in muscles of patients with CPT II deficiency are reported to be undetectable [30,63,64], reduced [62,63,64,65,66,67,68] or normal [69].

## 6. Recombinant Enzyme Studies

Recently, His6-N-hCPT2 (wild type) and His6-N-hCPT2/S113L (variant) were produced recombinantly in prokaryotic host in our laboratory. The recombinant enzymes were then purified and characterized according to their functional and regulatory properties [71]. Our findings revealed impaired kinetic stability of human CPT II by the common p.Ser113Leu mutation at increased temperatures [62]. The observation was consistent with the lower heat resistance of the mutated enzyme in cultured fibroblasts [72]. These results show that CPT II is extremely thermoliable and abnormally inhibited. The biochemical consequences of other CPT II mutations in our laboratory are still under investigation.

## 7. Fibroblast Growth Factor 21 (FGF-21) Mitochondrial Biomarker

FGF-21 has been established as a potential biomarker for diagnosis of mitochondrial diseases [73,74,75,76,77]. The serum FGF-21 concentrations that were measured in 13 CPT II deficient patients were, however, not significantly different from those of controls [78]. This was independent of the underlying mutation, gender, body-mass index (BMI), and number and severity of attacks. A previous study has proposed induction of FGF-21 in mitochondrial myopathies to be a transcriptional response which is suggested to be a part of an integrated mitochondrial stress response [79]. Additionally, it has been shown that the mammalian target of rapamycin (mTOR)-regulated genes were significantly upregulated in a mouse model with cardiac and muscle CPT II deficiency (Cpt2M−/−) [80]. Serum FGF-21 level was also significantly increased above normal level in CPTII knock out mice as well [78,79,80,81,82,83,84,85]. Based on these findings, a pathogenically higher FGF-21 serum level in patients with muscle CPT II deficiency can be expected. However, this was not the case in our patients. It has to be noted that the serum samples in our patients were collected during attack-free intervals. This further strengthens the notion that in muscle CPT II deficient patients, there is no permanent lack of active enzyme but rather an abnormal regulation and thermostability of the mutant enzyme [29,61,62].

## 8. Genotype-Phenotype Analysis

A possible phenotype/genotype correlation in CPT II deficiency has been suggested in some studies. Mutations such as p.Pro227Leu, c.1923_1935del, Asp328Gly on both alleles are reported exclusively in neonatal or severe infantile form [20,21]. These mutations result in severe symptoms leading to death. These severe mutations are generally not found in patients with mild muscle CPT II deficiency. However, severe infantile form of CPT II deficiency is also described in compound heterozygous states in combination with a severe pathogenic variant and a variant usually associated with mild phenotype (p.Gly520Ala) [22]. Apart from that, the p.Arg503Cys pathogenic variant was identified in a family presented with a slowly progressive mild myopathy characterized by progressive muscle weakness and myopathic symptoms [16]. Moreover, isolated cases of myopathy related to *CPT2* gene mutations have also been reported [86]. Not only pathogenic mutations but the polymorphisms have also been identified to be detrimental. The polymorphism p.Phe352Cys has been reported to be associated with acute encephalopathy during infectious disease and sudden unexpected death in infancy in East Asian population. This polymorphism is thermolabile and reduces enzyme activity during high temperatures [87,88].

On the other hand, the most frequent p.Ser113Leu mutation associated with mild muscle form is not found in other severe forms of CPT II deficiency. The p.Ser113Leu is frequently found in northern Europeans while p.Phe383Tyr appears to have the highest prevalence in Japanese population [89].

A genotype-phenotype correlation analysed based on patients with homozygous p.Ser113Leu mutation and patients with heterozygous p.Ser113Leu mutation in our cohort of 50 patients revealed similar frequencies of the age of onset, frequencies of symptom of attacks and triggering factors were similar. There was no significant difference in the residual activity upon inhibition with Triton X-100 in both groups [29]. Hence, a clear genotype-phenotype correlation in muscle CPT II deficiency is not known.

## 9. Manifesting Heterozygotes

**Figure 4:**
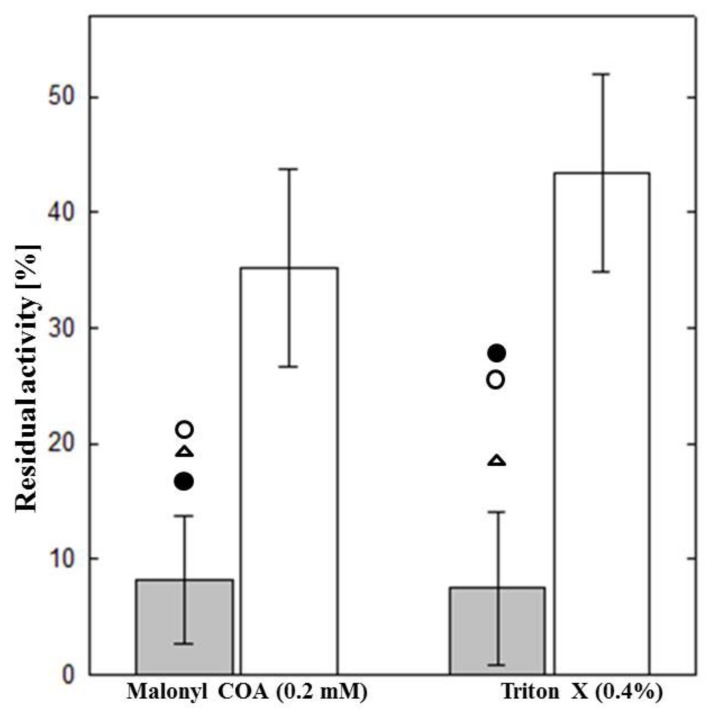
Residual CPT Activity (% of total CPT I and II) after pre-incubation with malonyl-CoA and Triton-X. Grey bars represent patients with mutations on both alleles (*n* = 40) and white bars represent healthy controls (*n* = 21). The error bars represent standard errors. Three manifesting heterozygotes patients are represented by closed circle, open circle and open triangle, respectively.

We had previously reported two manifesting heterozygote patients [90]. Apart from those two, recently we have identified one more clinically symptomatic heterozygote patient in our department. All three patients showed typical symptoms of muscle CPT II deficiency: exercise-induced attacks of muscle weakness and onset in adulthood. The intermediate residual enzyme activities after pre-incubation with Triton X-100 and upon addition of malonyl- CoA are consistent with the molecular finding of only one mutation in these patients (Figure 4).

Although CPT II deficiency is considered to be an autosomal recessive disorder, these three cases indicate that heterozygotes with only one mutant allele might also show the typical attacks of symptoms. However, all three patients were professional athletes and the attacks took place after strenuous physical activities (such as after a game of tennis, running a 10 km distance on a hot sunny day, after working hard the whole afternoon in garden). These are the typical triggers for inhibition of the enzyme [29,33]. Hence, it would be really interesting to document physical activities of further heterozygotes to identify a possible cut-off (duration of physical activity, severity of the activity, CK, myoglobin, etc.) for emergence of attacks. These observations strengthen that despite the recessive mode of inheritance of this disorder; the heterozygotes could not only be regarded as asymptomatic carriers but could also occasionally show typical symptoms. Hence, if CPT II deficiency is identified in any member in the family, the mutation carriers in the family may also be at risk of attack. Genetic counselling will be helpful to them.

## 10. Treatment

There is no approved drug for treatment of CPT II deficiency. Complete body rest and carbohydrate supplement seem to diminish the severity of symptoms during and after the attacks [33]. Therefore, the patients are advised to avoid strenuous physical activities. A carbohydrate rich nutrient with low fat content and carnitine supplement is also suggested. Shifting nutrient habit in this direction improves the acylcarnitine profile that eventually avoids risk of further attacks of hypoglycaemia and arrhythmia [91]. Moreover, in muscle CPT II deficient patients, complication during general anaesthesia (including rhabdomyolysis and renal post-anaesthetic failure) has also been reported [92,93]. Hence, it is advised to evaluate the asymptomatic at-risk relatives, as well. Early diagnosis and precautions can immensely reduce the possible fatality caused by CPT II deficiency.

A couple of clinical studies are underway towards finding potential therapy for CPT II deficiency. Previously, Roe and colleagues were able to revert rhabdomyolysis in seven CPT II deficient patients after anaplerotic diet therapy [94]. These individuals returned to normal physical activity including strenuous sports after this therapy. Moreover, another study demonstrated the increase of *CPT2* mRNA and normalization of enzyme activity in mild forms of CPT II-deficient cultured fibroblasts and myoblasts by bezafibrate treatment [95]. Furthermore, a trial on six CPT II deficient patients that were treated with bezafibrate showed elevation of fatty acid oxidation levels in muscle biopsies as well as a significant increase in palmitoyl-L-carnitine oxidation, increased CPT2 mRNA, and increased translated protein. In these patients, episodes of rhabdomyolysis were considerably decreased and the quality of life (measured by SF-36) analysis showed increase in physical activity and a decline in intensity of muscle pain [95]. Despite these steps put forward towards finding diagnosis and management strategies of CPT II deficiency, this muscle disorder is still a mystery for many medical doctors, clinical geneticists and even for the patients. A questionnaire-based study that we performed showed that it took average of 26.7 years to get diagnosed that involved several physicians [33]. However, the knowledge and awareness of CPT II deficiency is gradually increasing among patients and clinicians and in due time more and more cases of CPT II deficiency are bound to emerge. This will also pave the path towards finding a potential therapy in this metabolic disorder.

## 11. Conclusions and Summary

Studies on CPT II deficiency emphasize that they are the most common disorders of long-chain fatty acids. However, only around 400 unrelated families with different three forms of CPT II deficiency have been reported globally. It can be predicted that there should be a lot more undiagnosed cases of CPT II deficiency as the patients are suffered by occasional attacks of symptoms. Between the attacks, the patients are generally normal and can perform their daily activities without much problem. Additionally, females seem to be less likely to develop myoglobinuria and therefore remain undetected. The diagnostic facility of CPT II deficiency is not readily available and lots of patients have to wait for multiple decades just to get the proper diagnosis. Molecular genetic investigation is regarded as the gold standard in diagnosis of CPT II deficiency. Due to the introduction of multi-panel gene sequencing of muscle patient and exome sequencing in recent times, new cases of CPT II deficiency are emerging more frequently. However, even during molecular genetic analysis, the potentially pathogenic silent mutations seem to be overlooked not only in CPT II deficiency but in a lot of autosomal disorders. In addition, biochemical analysis of muscle homogenates also potentially identifies CPT II deficient patients with high specificity. The MRI analysis of muscles of CPT II deficient patients or histological or immunohistological analysis are not conclusive and cannot be accepted as potential diagnostic measures in CPT II deficiency. The MRI of affected muscle of CPT II deficient patients during an attack would be really interesting to analyse. However, this does not seem to be feasible as the patients suffer from unexpected attacks of symptoms. Unlike enzyme replacement therapy in some metabolic disorders such as late onset Morbus Pompe, there is no therapy available for CPT II deficiency. Complete body rest, supplement of carbohydrate rich nutrition and substitution of fluids seem to decrease the frequency and intensity of attacks.

## Figures and Tables

**Table 1:** List of known mutations in *CPT2* Gene. Mutations identified in our cohort are marked red, novel mutations identified in our department are in bold cases. Intronic mutations are marked with asterisk. Intronic mutations other than those identified in our cohort are marked blue. Severe mutations reported exclusively in neonatal and infantile form are marked brown. All the mutations are listed on Human Gene Mutation Database (HGMD) [70].

**Exon 1**	**Exon 4**
p.Pro41Leu p.Pro50His c.36-38 insGC c.36_43dupGGGCCC c.113_114dupGC	p.Tyr120Cys p.Leu121Gln p.Arg124Gln p.Arg124Ter p.Asn146Thr p.Arg151Gln p.Arg151Trp p.Arg161Trp p.Lys164Ter p.Arg167Gln p.Pro173Ser p.Glu174Lys p.Tyr210Asp p.Asp213Gly p.Met214Thr p.Gln216Arg p.Pro227Leu p.Arg231Trp p.Arg247Trp p.Lys274Met p.Arg296Gln p.Arg296Leu p.Arg296Ter p.Gly310Gly p.Cys326Tyr p.Asp328Gly p.Met342Thr p.Phe352Cys p.Val368Ile p.His369Gln p.Arg382Lys p.Phe383Tyr p.Gln413Gln p.Phe448Leu p.Arg450Ter **p.Gly451Glu** p.Glu454Ter p.Lys457Ter p.Tyr479Phe p.Tyr479Cys p.Gly480Arg p.Glu487Lys p.Gly497Ser p.Ile502Thr p.Arg503Cys p.Pro504Leu p.Phe516Ser p.Glu545Ala c.1569_1570delCA c.1444_1447delACAG c.1634_1636delAAG **c.1646_49del** c.1273_1274delAC c.1238_1239delAG c.1543_1546delGCCT c.907_918ins11 c.533insT c.534-558del25 c.1645+5G>A (Intron 5)*
**Exon 2**	
p.Pro55Arg p.Ala67Gly ** c.182_203del 22** c.153-1G>A (Intron 2)*	
**Exon 3**	
p.Cys84Arg p.Ala101Val p.Ser113Leu c.256_257delAG c.232+1G>A (Intron 3)*	
**Exon 5**	
p.Arg560Gln p.Leu575Pro p.Asp576Gly p.Ser588Cys **p.Ser590Asn ** p.Gly600Arg p.Pro604Ser p.Val605Leu p.Asp608His p.Tyr628Ser p.Arg631Cys p.Leu644Ser c.1816_1817delGT 14. c.1923_1935del 15. c.340+1G>A (Intron 4)* 16. c.340+5G>A (Intron 4)*	
